# Parental Language Mixing in Montreal: Rates, Predictors, and Relation to Infants’ Vocabulary Size

**DOI:** 10.3390/bs15101371

**Published:** 2025-10-08

**Authors:** Alexandra Paquette, Krista Byers-Heinlein

**Affiliations:** Department of Psychology, Concordia University, 7141 Sherbrooke St. West, PY-033, Montréal, QC H4B 1R6, Canada; k.byers@concordia.ca

**Keywords:** language mixing, code-switching, bilingualism, vocabulary development

## Abstract

Language mixing is a common feature of bilingual communication, yet its predictors and effects on children’s vocabulary development remain debated. Most research has been conducted in contexts with clear societal and heritage languages, leaving open questions about language mixing in environments with two societal languages. Montreal provides a unique opportunity to examine this question, as both French and English hold societal status, while many families also maintain heritage languages. Using archival data from 398 bilingual children (7–34 months), we looked at French-English bilinguals (representing societal bilingualism) and heritage-language bilinguals within the same sociolinguistic environment. We assessed the prevalence, predictors, and motivations of parental language mixing and its relationship with vocabulary development. Results revealed that mixing was less frequent among French-English bilinguals compared to heritage-language bilinguals in the same city. The direction of mixing differed between groups: French-English bilinguals mixed based on language dominance, while heritage-language bilinguals mixed based on societal language status. Primary motivations included uncertainty about word meanings, lack of suitable translations, and teaching new words. Mixing showed minimal associations with vocabulary size across participants. These findings suggest that parental mixing practices reflect adaptive strategies that vary by sociolinguistic context rather than detrimental influences on early language acquisition.

## 1. Introduction

Language mixing—also called code-switching—involves incorporating words and phrases from one language while using another. This practice is commonplace in bilingual environments and represents a significant aspect of many children’s early language experiences (Bail et al., 2015; Byers-Heinlein, 2013; Kremin et al., 2022). In Canada, around 18% of children aged 0–9 years live in bilingual homes, with this percentage rising to over 27% in major metropolitan cities such as Montreal, Vancouver, and Toronto (Schott et al., 2022). Globally, it is estimated that at least half of the world’s population is bilingual (Grosjean, 2021; UNESCO, 2024). Given the prevalence of bilingualism, understanding why parents mix their languages and how this practice impacts children’s language development is crucial for supporting bilingualism across diverse environments. The current study investigated three key aspects of language mixing in French-English and heritage-language bilingual families in Montreal: (a) the prevalence and predictors of parental language mixing, (b) parents’ motivations for language mixing, and (c) links between exposure to language mixing and vocabulary development.

### 1.1. Sociolinguistic Context in Montreal

The sociolinguistic use of French and English in Montreal, Quebec, presents a distinctive context for studying bilingual language practices. Unlike many bilingual communities where one language holds a clear majority status, in Montreal both French and English function as societal languages, meaning they are broadly used across public life (Kircher et al., 2022). Approximately 92% of Montrealers know French, which is the official language of the province and one of Canada’s official languages, and 64% of Montrealers know English, which is Canada’s other official language (Statistics Canada, 2023a). Montreal has the second-highest rate of English-French bilingualism in the country (56.4%), after the Ottawa-Gatineau region (Statistics Canada, 2023b). The city’s bilingual character is evident in everyday life—commercial signage is often displayed in both English and French, and staff in stores and service outlets frequently use the “bonjour, hi” greeting, reflecting Montreal’s inherently bilingual nature (Leimgruber & Fernández-Mallat, 2021). This dual-language environment shapes children’s exposure to language mixing as they grow up in a setting where both English and French hold high status and are regularly encountered in daily interactions. Importantly, Montreal is also home to a large population of heritage-language speakers. Approximately 23% of residents report a mother tongue that is neither English nor French (Statistics Canada, 2023a), and 40% of students in the city’s public school system speak a heritage language—defined as a language other than the dominant languages in a given social context (Kelleher, 2010)—as their first language (D. J. Rowe, 2023). The most commonly spoken heritage languages in Montreal are Arabic (5.7%), Spanish (4.6%), and Italian (3.3%) (Statistics Canada, 2023a). In terms of household language use, 10.5% of Montreal residents only speak a heritage language, 6.4% speak English and a heritage language, 10% speak French and a heritage language, and 7.8% speak both French and English alongside a heritage language (Statistics Canada, 2023a). This makes Montreal a rich context for examining both societal and heritage bilingualism.

### 1.2. Parental Language Mixing in Montreal: Rates, Motivations, and Predictors

Different bilingual communities mix their languages to different degrees (Poplack, 1985). Studies using daylong home language recordings found that French-English bilingual parents in Montreal mixed languages at relatively modest rates—seven times per hour with 10-month-olds and 28 times per hour with 18-month-olds (Kremin et al., 2022). In contrast, studies of Spanish-English bilinguals in Maryland, U.S., recorded much higher rates, with parents mixing 30 times during brief 13 min play sessions. These differences may reflect distinct social motivations for language mixing. Evidence suggests that bilinguals mix languages to help build and maintain a sense of community (Nilep, 2006) and form social and group identities (Botha, 2021; Guerini, 2014; Pérez-Sabater & Moffo, 2019; Phinney et al., 2001). Consistent with this research, in Montreal, where both French and English are societal languages, there might be a reduced need for language mixing to maintain group identity among French-English bilinguals (Kircher, 2009), while heritage-language bilinguals in Montreal may rely more on language mixing as a strategy to reinforce cultural identity and maintain their heritage language.

While adult language mixing broadly serves social functions, research on parent–child interactions suggests additional pedagogical motivations for language mixing in this context. A longitudinal study found that Montreal parents speaking French and English mixed languages to help children learn vocabulary, and parental language mixing increased with the child’s age—parents mixed more to teach vocabulary when their infant was 18 months old compared to when they were 10 months old (Kremin et al., 2022). While motivations for language mixing among heritage-language bilingual parents have not been investigated in Montreal, the majority of Vancouver parents (52%) reported borrowing a word from a heritage language when speaking English to teach their children new vocabulary (Byers-Heinlein, 2013). These findings indicate that bilingual parents may strategically use language mixing as a pedagogical tool to support their children’s vocabulary development, particularly as children reach developmental stages where they are acquiring new words more rapidly (Bergelson, 2020).

Beyond pedagogical reasons, several other factors correlate with parental language mixing. Mixing occurs more frequently in bilingual households where each parent speaks both languages to their children, as compared to one-parent-one-language households where mixing within a single speaker is less likely to occur (Verhagen et al., 2022). Parents who use their languages across more diverse contexts (e.g., at home, at playgroup, when with family) with their children also report higher rates of language mixing (Byers-Heinlein, 2013). These patterns suggest that language balance and context diversity may be important predictors of mixing behaviors.

### 1.3. How Parental Language Mixing Relates to Word Learning and Vocabulary Development

Parents’ language practices significantly influence bilingual children’s vocabulary development and proficiency (De Houwer, 2007; Dijkstra et al., 2015; Gathercole & Thomas, 2009; Verhagen et al., 2022). Language acquisition depends on both input quantity and quality (Anderson et al., 2021; Schwab & Lew-Williams, 2016; Unsworth, 2016), and language mixing may affect input quality in complex ways.

M. L. Rowe and Snow (2019) identify three dimensions of input quality: linguistic, interactive, and conceptual. Language mixing might enhance the linguistic and interactive dimensions of input quality. First, linguistically, language mixing exposes children to more varied phonological, lexical, and grammatical structures across two languages. Second, language mixing in response to a child’s knowledge, needs, or interests could enhance interactional quality by making input more responsive and tailored—a key feature of high-quality input (M. L. Rowe & Snow, 2019)—particularly when performed to teach new words. However, mixing could also pose cognitive demands, particularly for younger infants who are still working to discriminate and separate their languages (Curtin et al., 2011). For example, some studies have found that language switching increases processing effort in adult bilinguals (Abutalebi et al., 2007; Liao & Chan, 2016; Tamargo et al., 2016). If language mixing causes processing delays or reduces clarity, it could detract from the child’s involvement in interactions with their parents. However, a recent study found adult bilinguals reported greater attention and memory when a story included language mixing (Salig et al., 2025), suggesting these additional cognitive demands may sometimes be beneficial.

Laboratory studies investigating the immediate effects of language mixing on language learning and processing have yielded mixed results. Bilingual toddlers understand familiar words more slowly in mixed-language sentences (Byers-Heinlein et al., 2017), particularly when switching into their non-dominant language (Potter et al., 2019). When examining novel word learning in mixed-language contexts, studies show inconsistent findings. French-English bilingual 3-year-olds in Montreal successfully learned a novel word when introduced in a single-language sentence (e.g., “Do you see the *dog* on the *teelo*?”) but struggled when it was introduced in a mixed-language sentence (e.g., “Do you see the *chien* [fr. dog] on the *teelo*?”), while Spanish-English bilinguals failed to learn in either context (Byers-Heinlein et al., 2022). By contrast, Kaushanskaya et al. (2022) found that 4-to-5-year-old children needed fewer exposures to learn new words introduced through mixed-language speech compared to English-only speech. Tsui et al. (2023) directly compared different types of language mixing and found that bilingual 3- to 5-year-olds learned new words equally well whether the switch between languages happened immediately or was delayed. Moreover, Libersky et al. (2025) demonstrated that bilingual 4- to 5-year-olds successfully learned both verbs and nouns in codeswitched contexts, modulated by their language skills and learning opportunities. In shared book reading contexts, language switching has been shown to either have no impact (Brouillard et al., 2022) or a positive impact on preschoolers’ word learning (Read et al., 2021).

Beyond immediate processing effects, researchers have examined how parents’ everyday language mixing relates to their children’s vocabulary development. The Language Mixing Questionnaire (Byers-Heinlein, 2013) has become a widely used tool for assessing parental language mixing frequency (Gross et al., 2022; Kremin et al., 2022; Place & Hoff, 2016; Potter et al., 2019). Using this measure, Byers-Heinlein (2013) found that 90% of bilingual Vancouver parents mixed languages when speaking to their children, and higher rates of mixing were associated with smaller English comprehension vocabularies in 1.5-year-old bilinguals, and marginally smaller production vocabularies in 2-year-old bilinguals, after controlling for English exposure.

However, other studies, using both similar and different methods, have reported diverging results. Verhagen et al. (2022) found no relationship between parents’ self-reported language mixing and 2–4-year-olds’ language proficiency in either Dutch or their heritage language. Similarly, Verhoeven et al. (2025) found no relation between parental language mixing and 3- to 5-year-old Turkish-Dutch and Polish-Dutch children’s outcomes in the majority language, when language mixing was measured using daylong recordings and a parent-report questionnaire. However, they did find a negative relationship with expressive vocabulary in the heritage language. In a natural-play study with English-Spanish toddlers and their caregivers, Bail et al. (2015) found that children whose parents mixed languages more had larger vocabularies, contradicting concerns about negative effects. Place and Hoff (2016) reported no relation between language mixing and 2-year-old toddlers’ language abilities when mixing was reported through language diaries, but found weaker Spanish skills in children whose parents reported more frequent mixing on questionnaires.

These inconsistent findings suggest that the relationship between parental language mixing and word learning and vocabulary development is complex and potentially influenced by multiple factors, including child age, sociolinguistic context, measurement approaches, and other factors unique to specific bilingual communities.

### 1.4. Our Studies

This project investigated language mixing in the unique bilingual context of Montreal, Quebec. While previous research has focused on bilingual communities with a societal language and one or more heritage languages—such as Spanish-English bilingual families in the United States (Bail et al., 2015), and bilingual families in Vancouver where English dominates (Byers-Heinlein, 2013) or bilingual families in the Netherlands, where Dutch is the societal language (Verhagen et al., 2022; Verhoeven et al., 2025)—Montreal presents a distinct sociolinguistic environment where both French and English function as societal languages (Kircher et al., 2022). At the same time, a sizable proportion of the population speaks a heritage language in addition to the societal languages. This setting allows for a unique comparison between societal-language bilingualism (i.e., French-English) and heritage-language bilingualism within the same community.

Across two studies, we used the Language Mixing Questionnaire (LMQ; Byers-Heinlein, 2013), a validated parent-report measure that assesses the frequency and motivations of language mixing in bilingual families, and allows for direct comparison with findings from other bilingual communities that have employed the same measure. In Study 1, we examined French-English bilingual families, while in Study 2, we focused on heritage-language bilingual families who were proficient in a heritage language and in English and/or French.

## 2. Study 1: Parental Language Mixing Amongst French-English Bilinguals

We analyzed data from 312 parents of French-English bilingual children aged 7–34 months in Montreal. This pre-registered study aimed to characterize language mixing practices in a community with two societal languages, and to compare these patterns with those observed in other bilingual populations.

### 2.1. Research Questions and Hypotheses

We first examined whether parental language mixing amongst French-English bilingual parents in Montreal constitutes a unitary construct. This analysis served to further validate the questionnaire and ensure that its psychometric properties were consistent in a different bilingual community from the original validation sample in Vancouver (Byers-Heinlein, 2013). We predicted that language mixing among French-English bilingual parents in Montreal would be a unitary construct, as measured by the items on the Language Mixing Scale.

Next, we investigated the frequency of language mixing amongst French-English bilingual parents in Montreal compared to other bilingual communities. Based on previous research (Kremin et al., 2022), we predicted that while most parents would report some language mixing, rates would be lower than those observed in Vancouver (Byers-Heinlein, 2013) and among Spanish-English bilinguals in the U.S. (Place & Hoff, 2016; Potter et al., 2019).

We also explored whether language mixing rates would differ when parents switched from the dominant to their non-dominant language or vice versa. We anticipated that parents would switch from their non-dominant to dominant language more often than the reverse, given research showing that switching to one’s non-dominant language requires greater cognitive effort (Liao & Chan, 2016). We additionally conducted exploratory analyses examining whether mixing patterns differed when switching from French to English or vice versa.

Our next research question focused on why French-English bilingual parents mix languages, and whether their motivations change as children develop. We predicted that the most common reasons would include teaching new vocabulary and addressing translation gaps, similar to previous findings (Byers-Heinlein, 2013; Kremin et al., 2022). Following Kremin et al. (2022), we predicted that parents would increasingly report using language mixing to teach vocabulary as children grew older.

We then investigated predictors of parental language mixing. We predicted that more balanced use would correlate with increased mixing, consistent with prior research (Byers-Heinlein, 2013; Verhagen et al., 2022). We further predicted that parents of older children would mix more frequently (Kremin et al., 2022), and that parents using their languages across more contexts would report higher mixing rates (Byers-Heinlein, 2013).

Finally, we examined the relationship between parental language mixing and children’s vocabulary size. Given conflicting findings from previous studies—showing positive (Bail et al., 2015), negative (Byers-Heinlein, 2013; Place & Hoff, 2016), or null relationships (Verhagen et al., 2022; Verhoeven et al., 2025)—we approached this question exploratively without strong directional hypotheses.

### 2.2. Methods

This study used archival data collected when children participated in various experimental studies conducted at the Concordia Infant Research Lab from 2011 to 2024. All studies were approved by the Human Research Ethics Board of Concordia University. The current study design and data analysis plan were pre-registered at https://osf.io/3g6ze/ (accessed on 18 December 2024). Any deviations from the pre-registration are listed and justified in the Supplemental Materials. All analyses were performed in the open-source statistical language R (R Core Team, 2021) within RStudio (version 2022.07.1+554), using the lmerTest (Kuznetsova et al., 2013), psych (Revelle, 2007), and lavaan (Rosseel et al., 2012) packages.

#### 2.2.1. Participants

The sample in Study 1 was contributed by 312 French-English bilingual children (yielding 389 data points as some participants contributed data twice) aged 7–34 months (*M* = 19.61, *SD* = 7.17; 50% female) and their parents. Participants were recruited from a database of interested families in Montreal, Canada, and had been recruited via provincial birth lists, at libraries and community events, as well as via social media advertisements. Families visited our laboratory between 2011 and 2024 to participate in various in-person experimental studies, during which they completed the questionnaires analyzed in this study.

Inclusion criteria were the following: full-term pregnancy (i.e., at least 37 weeks of gestation), normal birth weight (>2500 g), and no reported developmental delays or major health issues. Bilingual children were defined as those exposed to each English and French for at least 10% and at most 90% of the time over the course of their lives since birth, with less than 10% of exposure to a third language. On average, participants were exposed to English 53% of the time, and to French 47% of the time. While 51% of parents predominantly spoke English with their child, 43% predominantly spoke French, and 5% reported speaking an equal amount of French and English (these parents were excluded from analyses involving dominant/non-dominant classifications).^1^ On average, parents had a balance score of 18.93 (*SD* = 14.85), which reflects the percentage of time a parent spoke their less-used language to their child (e.g., a parent who spoke English 70% of the time and French 30% would have a balance score of 30, while a parent who spoke each language equally would have a score of 50). Higher balance scores indicate more balanced use of both languages. Parents completed the Language Experience Questionnaire (LEQ), Language Mixing Questionnaire (LMQ), and the MacArthur-Bates Communicative Development Inventories (CDIs) for both French and English, which are described in further detail below.

#### 2.2.2. Measures

Language Experience Questionnaire (LEQ). The LEQ (Bosch & Sebastián-Gallés, 2001) is a structured interview that assesses the infant’s language environment and yields a percentage estimate of their relative exposure to each of their languages from birth up until the day of assessment. It was used in conjunction with the Multilingual Approach to Parent Language Estimates (MAPLE; Byers-Heinlein et al., 2019) to maximize the quality of parents’ reports.

Language Mixing Questionnaire (LMQ). The LMQ (Byers-Heinlein, 2013) is a self-report measure designed to assess the frequency of mixing of two languages in bilingual parents’ speech to their children. The questionnaire includes background questions about parents’ language use, such as the contexts in which they speak their first and second language with their child and the percentage of interactions conducted in each language. From this, we derived parents’ balance scores and determined their dominant language based on the language they used most with their child.

To measure language mixing, the LMQ includes five Likert-scale items that assess how frequently parents engage in language mixing behaviors. For French-English bilinguals, these items include: (1) “I often start a sentence in English and then switch to speaking French,” (2) “I often start a sentence in French and then switch to speaking English,” (3) “I often borrow a French word when speaking English,” (4) “I often borrow an English word when speaking French,” and (5) “In general, I often mix English and French.” Respondents answer these items on a Likert scale from 1 (very true) to 7 (not at all true). All items are then re-coded on a 0–6 scale so that a higher score indicates a higher frequency of language mixing (i.e., 0 = “Not at all true”, 3 = “Somewhat true”, and 6 = “Very true”).

For items 3 and 4, follow-up questions probe the reasons for borrowing words, asking parents to select from the following options: (a) “I’m not sure of the word in the other language,” (b) “No translation or only a poor translation exists for the word,” (c) “The word is hard to pronounce,” (d) “When I’m teaching new words,” or (e) “Other times/not sure.”

For some analyses, we recoded the five Likert items to reflect the frequency of language mixing from each parent’s dominant language to their non-dominant language and vice versa. This recoding approach is informed by research indicating that language mixing behavior is influenced by an individual’s relative language dominance (e.g., Liao & Chan, 2016). This recoding complements the original coding, which reflects mixing between English and French, allowing us to examine whether the underlying construct of language mixing is consistent across different language groupings.

The responses to the five Likert-scale items are added to create a Language Mixing Scale score, which ranges from 0 (no language mixing) to 30 (frequent language mixing). The scale has demonstrated high reliability in a sample of English-other bilinguals in Vancouver (α = 0.84; Byers-Heinlein, 2013). All parents completed the LMQ in their preferred language (in English or in French). In most cases, one parent completed the LMQ (typically the primary caregiver), but in cases where two parents did, we retained the data from the mother or other indicated primary caregiver.

MacArthur-Bates Communicative Development Inventories (CDIs). The MacArthur Bates CDI is a parent-report instrument assessing children’s expressive and receptive vocabularies. CDIs are available in many languages, including Canadian French (Trudeau et al., 1999) and American English (Fenson et al., 2007), which all children in this study were acquiring. Parents completed two CDIs for their children—one in English and one in French. Whenever possible, the parent who was most familiar with the child’s vocabulary in that language completed the form. Parents of children who were 16 months old and younger filled out the Words and Gestures form of the CDI, which asks about both word comprehension and word production. Parents of children older than 16 months filled out the Words and Sentences form of the CDI, which asks only about word production.

There are several different ways that bilingual children’s vocabulary size can be measured. Combined vocabulary scores can be calculated for either concepts or words (Byers-Heinlein et al., 2024; Pearson et al., 1993). Word vocabulary measures the total number of words the child knows in each of their languages, while concept vocabulary measures the number of concepts the child knows in either language (Byers-Heinlein et al., 2024). Single-language vocabulary sizes can also be calculated, either by language (French vs. English) or by language dominance (dominant vs. non-dominant language). Moreover, each of these metrics could be examined in receptive or in productive vocabulary. Our approach was to look at whether any of these different ways of measuring vocabulary would be related to language mixing. Thus, we calculated a total of 12 different possible vocabulary outcome variables.

### 2.3. Results

#### 2.3.1. Language Mixing Scale Validity in Montreal

We first examined the psychometric properties of the Language Mixing Scale to validate its use with French-English bilingual parents in Montreal, a population that differs from the original validation sample in Vancouver (Byers-Heinlein, 2013). Our analysis revealed similar psychometric properties whether items were coded to reflect mixing between dominant and non-dominant languages or specifically between French and English. For simplicity, we report results for coding based on language dominance (see Supplemental Materials and Tables S1–S3 for additional analyses).

Spearman’s rank correlations between all five Likert items were significant (*r*s = 0.39–0.62, all *p*s < 0.001). An exploratory factor analysis examining the scale’s underlying factor structure revealed a primary component with an eigenvalue of 3.10, accounting for 51% of the variance. All other eigenvalues were under 1, suggesting a one-factor solution. Parallel analysis confirmed that a single factor should be retained, as both the number of factors and components were estimated to be 1. Inter-item correlations and extracted loadings of all the items on the factor are reported in Table 1. The scale demonstrated good internal consistency, with a Cronbach’s alpha of α = 0.84, consistent with the findings from the Vancouver sample reported by Byers-Heinlein (2013).

These results indicate that the Language Mixing Scale is a valid measure of the unitary construct of language mixing in this sample of Montreal French-English bilinguals and, thus, it was psychometrically appropriate to calculate a Language Mixing Scale score for each parent as the sum of the responses across the five scale items. Mixing scores, therefore, ranged from 0 (no mixing) to 30 (highest amount of mixing).

#### 2.3.2. Prevalence of Language Mixing in Montreal and Comparisons to Other Communities

**Figure 1:**
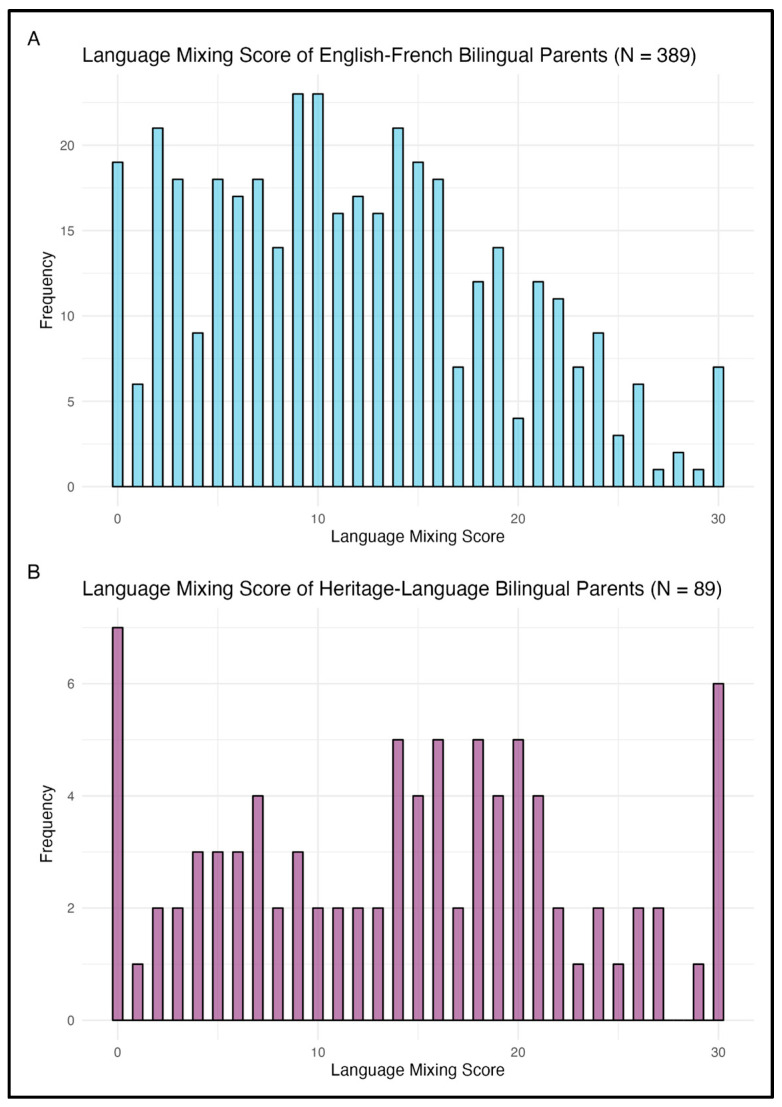
Language Mixing Scale scores distribution across (**A**) French-English and (**B**) heritage-language bilingual participants. The x-axis represents the Language Mixing Scale score, which ranges from 0 (no language mixing) to 30 (frequent language mixing), while the y-axis shows the number of parents who reported each score on the Language Mixing Questionnaire (LMQ).

After validating the Language Mixing Scale, we examined the frequency of language mixing among French-English bilingual parents in Montreal and compared these rates to other bilingual communities. Across our sample, French-English bilingual parents had a mean mixing score of 11.74 (*SD* = 7.42) out of a possible 30, and the distribution was somewhat right-skewed, indicating that most parents clustered at lower mixing frequencies with fewer parents reporting very high levels of mixing (see Figure 1A).

Using two-sample, two-tailed *t*-tests, we found that language mixing amongst parents in Montreal was significantly lower than in Vancouver parents whose children ranged from 18 to 26 months (Byers-Heinlein, 2013; *M* = 13.30, *SD* = 7.80; *t*(336) = 2.26, *p* = 0.024, *d* = 0.21). Similarly, Montreal parents mixed less than parents of Spanish-English bilingual 30-month-old children in South Florida (Place & Hoff, 2016; *M* = 14.00, *SD* = 7.41; *t*(75) = 2.17, *p* = 0.033, *d* = 0.31), and parents of Spanish-English bilingual 18–30-month-old children in New Jersey (Potter et al., 2019; *M* = 17.00, *SD* = 8.60; *t*(18) = 2.55, *p* = 0.020, *d* = 0.66).

**Figure 2:**
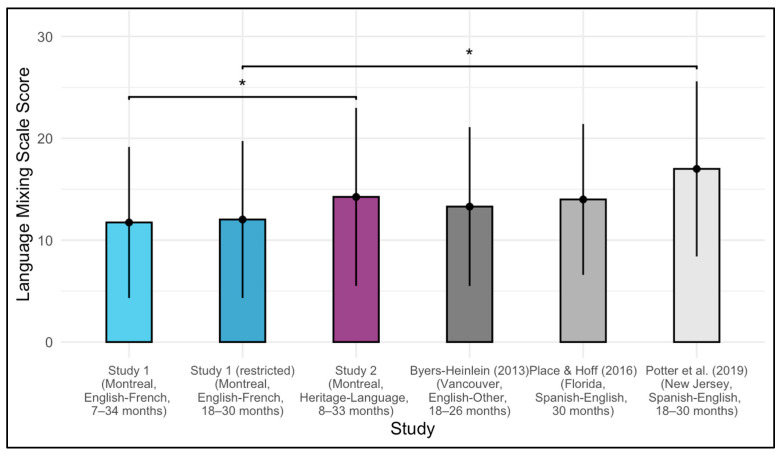
Language Mixing Scale scores across studies (Byers-Heinlein, 2013; Place & Hoff, 2016; Potter et al., 2019). The y-axis depicts the Language Mixing Scale score, which ranges from 0 (no language mixing) to 30 (frequent language mixing). Vertical lines represent one standard deviation above and below the mean in each sample. Horizontal lines indicate statistically significant comparisons based on age-matched samples (* *p* < 0.05). Although additional comparisons were conducted for the unrestricted Study 1 sample, these are not shown in the figure as they did not account for child age.

To account for potential age differences between samples, we next restricted our data to only include children aged 18–30 months (*n* = 180) and re-ran the two-sample, two-tailed *t*-tests. In this restricted sample, Montreal parents’ language mixing (*M* = 12.03, *SD* = 7.71) was no longer significantly less than that of parents in Vancouver (Byers-Heinlein, 2013; *t*(359) = 1.55 *p* = 0.122, *d* = 0.16), or South Florida (Place & Hoff, 2016; *t*(100) = 1.74, *p* = 0.086, *d* = 0.26). However, Montreal parents’ mixing was still significantly less than that of parents in New Jersey (Potter et al., 2019; *t*(20) = 2.36, *p* = 0.030, *d* = 0.61; see Figure 2).

In sum, after accounting for age, Montreal parents’ language mixing rates were largely comparable to those reported in other bilingual communities, with a significant difference emerging only in comparison to New Jersey parents.

#### 2.3.3. Parental Mixing Patterns

Beyond overall mixing rates, we examined the specific patterns in how parents mix their languages, focusing on directionality of mixing between languages. Using paired samples *t*-tests, we analyzed whether parents reported different frequencies of mixing depending on the direction of the language switch, both in terms of language dominance and specific languages.

When coded relative to parents’ language dominance, parents reported switching from their non-dominant to dominant language (*M* = 2.10, *SD* = 1.83) more often than from their dominant to non-dominant language (*M* = 1.93, *SD* = 1.81), although this difference was small and only marginally significant, *t*(367) = 1.86, *p* = 0.064, *d* = 0.09. However, parents reported borrowing words from their dominant language when speaking their non-dominant language (*M* = 2.64, *SD* = 1.98) significantly more often than borrowing words from their non-dominant language when speaking their dominant language (*M* = 2.32, *SD* = 1.84; *t*(367) = 3.25, *p* = 0.001, *d* = 0.16).

In analyses focused on specific languages rather than dominance, parents reported switching to French when speaking English (*M* = 2.23, *SD* = 1.89) significantly more often than switching to English when speaking French (*M* = 1.89, *SD* = 1.76; *t*(388) = 4.01, *p* < 0.001, *d* = 0.19). Conversely, parents reported borrowing an English word when speaking French (*M* = 2.59, *SD* = 1.94) significantly more often than borrowing a French word when speaking English (*M* = 2.38, *SD* = 1.90; *t*(388) = 2.26, *p* = 0.024, *d* = 0.11).

These findings reveal meaningful asymmetries in how bilingual parents mix their languages. While the tendencies were small to moderate in effect size, they consistently showed that parents engage in different mixing behaviors depending on which language they are primarily speaking.

#### 2.3.4. Motivations for Language Mixing and Relations to Child Age

We next examined parents’ motivations for language mixing, particularly their reasons for borrowing words from another language. When borrowing from their dominant language while speaking their non-dominant language, the most common reason was uncertainty about the word (63% of parents). When borrowing a word from their non-dominant language while speaking their dominant language, the primary reasons were the lack of a translation or a poor translation (53%) and teaching new words (53%).

For language-specific borrowing, 51% of parents reported borrowing English words when speaking French because of the absence of a suitable translation, while 49% said they were unsure of the French equivalent. French words were most often borrowed when teaching new words (50%) or due to the lack of a proper translation (48%). See Table 2 for the full proportions of parents reporting each reason for borrowing words.

These findings suggest that language dominance influences parents’ borrowing behavior due to uncertainty about words, while translation gaps and the desire to teach new words remain consistent reasons for borrowing across both languages, irrespective of parents’ dominant language.

To examine how motivations for language mixing might change as children develop, mixed-effects logistic regression models were fit with child age (in years) as a predictor for each potential reason for language mixing. In each model, the binary outcome variable indicated whether a parent selected that reason (1 = selected, 0 = not selected), and child age in years was entered as a fixed effect predictor. A random intercept for participant was included. Child age significantly predicted only one motivation: parents of older children were more likely to borrow a word from another language to teach new words, *p* = 0.014, *OR* = 1.83, 95% CI [1.14, 3.03]. This corresponded to an 83% increase in odds of borrowing for teaching purposes with each additional year of age. This developmental pattern suggests that as children grow older, parents increasingly employ language mixing as an intentional pedagogical strategy for vocabulary instruction rather than simply as a response to their own language limitations (all *OR*s < 1.08, *p*s > 0.195).

#### 2.3.5. Predictors of Language Mixing

We next examined the factors that predict the frequency of parental language mixing. Pearson correlations showed that the Language Mixing Scale scores were positively correlated with both the parent balance scores (*r* = 0.47, *p* < 0.001) and the number of contexts in which both languages were used (*r* = 0.26, *p* < 0.001). Parents with more balance in language use and those who used both languages in more diverse contexts engaged in more language mixing. No significant correlation was found between language mixing and child age (*r* = 0.07, *p* = 0.194).

To examine the relative contribution of these factors, a multiple linear regression was conducted predicting language mixing from parent balance score, number of contexts, and child age in years (see Table 3). The overall model was significant, *F*(3, 385) = 38.86, *p* < 0.001, with an *R*^2^ of 0.226. Two significant predictors emerged in this multivariate analysis: parents who reported more language mixing were more balanced in their bilingual language use with their child, and had older children. However, the number of contexts did not significantly predict language mixing when controlling for these other factors.

These findings suggest that parental language balance—the degree to which parents use both languages relatively equally with their child—is the strongest predictor of language mixing. The significant relationship with child age in the multivariate model, despite no significant bivariate correlation, suggests that age may interact with other factors in predicting language mixing behavior.

#### 2.3.6. Parental Language Mixing’s Relationship to Children’s Vocabulary Size

Finally, we examined the relationship between language mixing and children’s vocabulary development. We employed multiple vocabulary metrics to provide a comprehensive assessment, examining 12 possible vocabulary outcome variables: combined vocabulary scores for both concepts and words, single-language vocabulary sizes by language (French vs. English) and by language dominance (dominant vs. non-dominant), each measured in either receptive or productive vocabulary. We used a significance threshold of *p* < 0.05 and also applied a Bonferroni correction to account for multiple comparisons across vocabulary measures.

We first calculated Pearson pairwise correlations between language mixing scores and each vocabulary metric. These analyses revealed no significant association between any of the vocabulary metrics and language mixing, with the exception of English production vocabulary, *r* = 0.12, *p* = 0.019, which showed a weak but statistically significant correlation. However, this correlation would not survive correction for multiple comparisons. Additionally, these pairwise correlations are challenging to interpret because they do not account for age or language exposure—two factors that varied widely in our sample and are well-established predictors of vocabulary size.

Thus, we next conducted linear mixed-effects models with each vocabulary size metric as the outcome variable, and predictors including Language Mixing Scale score, age, and their interactions. Additionally, for single-language models (English, French, dominant, non-dominant), we included language exposure with respect to the measured language to account for the known relation between single-language vocabulary and exposure. We included a random intercept for participants (See Table S4 in Supplemental Materials for coefficients of all linear mixed effects models predicting vocabulary).

For all outcome variables except for English comprehension, there was a significant effect of age, such that older children knew more words. Amongst the models that included exposure (i.e., the single-language models), children with greater exposure had larger vocabularies except for the dominant language comprehension and dominant language production outcomes (i.e., exposure was significant for both comprehension and production for non-dominant language, English, and French).

**Figure 3:**
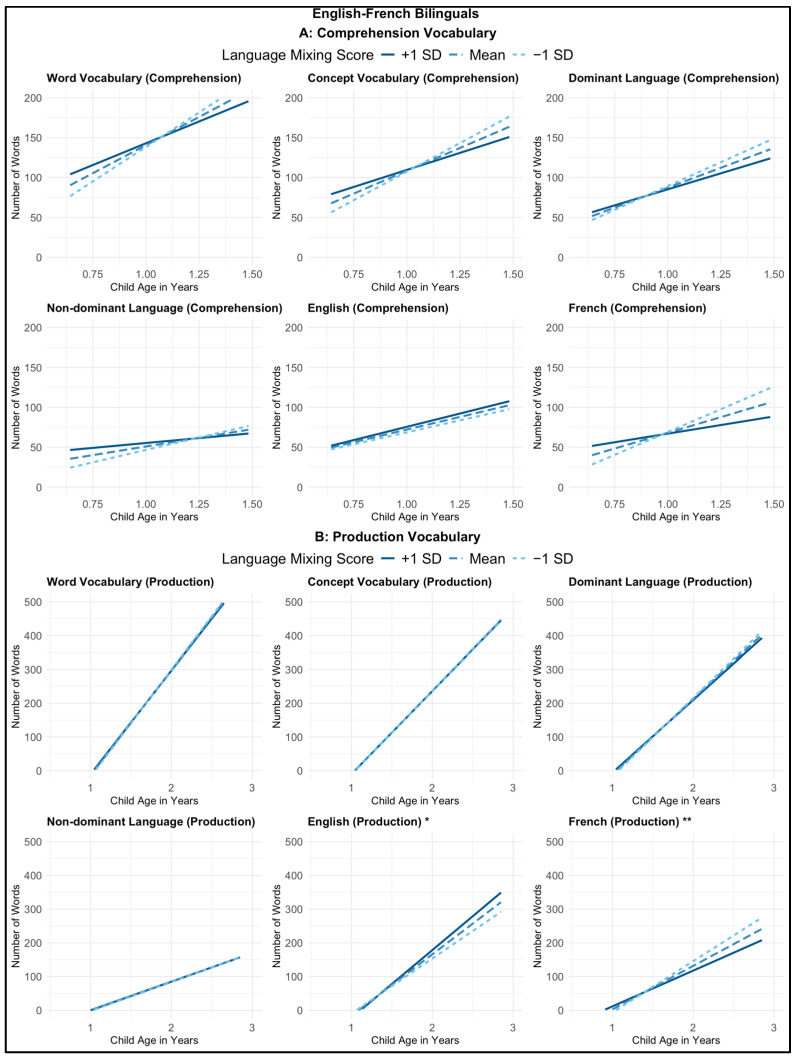
Interaction between child age and language mixing score on vocabulary development among French-English bilinguals, for (**A**) comprehension vocabulary measures and (**B**) production vocabulary measures. The x-axes represent child age in years, and the y-axes represent the number of words understood (**A**) or spoken (**B**). Each graph shows predicted vocabulary growth with age for children whose parents’ Language Mixing Scale score was at the sample mean (dashed line), 1 standard deviation above the mean (solid line), and 1 standard deviation below the mean (dotted line). * *p* < 0.05, ** *p* < 0.01 indicate significant interactions between child age and language mixing score.

Turning to our main interest, which was the effects of language mixing, in 10 of the 12 models, we found no main effect or interactions of language mixing with age in years. However, in two of the models, we found a significant age-by-language-mixing interaction. In the English production model, amongst older children, more language mixing was associated with a *larger* English productive vocabulary (*B* = 2.55, β = 0.02, *p* = 0.041), controlling for language exposure. Oppositely in the French production model, amongst older children, more language mixing was associated with a *smaller* French productive vocabulary (*B* = −3.10, β = −0.03, *p* = 0.002), controlling for language exposure. Note that only this latter finding survived correction for multiple comparisons (See Figure 3).

Our findings suggest that parental language mixing is largely unrelated to French-English bilingual children’s vocabulary development, with the exception of small, age-dependent effects on English and French productive vocabulary, which were in opposite directions.

### 2.4. Discussion

Study 1 examined parental language mixing among French-English bilingual families in Montreal, a unique context where both languages hold high sociolinguistic status. We validated the LMQ (Byers-Heinlein, 2013) in this population, showing it had strong internal consistency. Parents’ mixing was comparable to other bilingual communities, with parents only mixing less frequently than Spanish-English bilinguals in New Jersey. The direction of mixing was influenced both by language dominance and the specific language pair. Common motivations included uncertainty and translation gaps, as well as pedagogical goals such as teaching new words—particularly among parents of older children. Mixing behavior was best predicted by parents’ balance in language use. Language mixing was largely unrelated to vocabulary development, with only modest effects on French and English production vocabulary. These findings raise the question of whether observed patterns reflect broader features of the Montreal context or the status of French and English specifically, motivating our second study with heritage-language bilingual families.

## 3. Study 2: Parental Language Mixing Amongst Heritage-Language Bilinguals

Building on Study 1, this study examined language mixing among heritage-language bilingual parents in the same city, within the same sociolinguistic context. By comparing this group to the French-English bilinguals in Study 1, we aimed to better understand how community and language background influence parental language mixing practices. As such, we explored the same research questions as in Study 1 using data from 86 parents of heritage-language bilingual children aged 8–33 months.

### 3.1. Research Questions and Hypotheses

We first examined how often heritage-language bilingual parents in Montreal mix languages, and compared this to the French-English bilinguals from Study 1 as well as bilinguals from other communities. Based on the findings of Study 1 as well as previous research (Kremin et al., 2022), we predicted that the Montreal heritage-language bilinguals would mix more than French-English bilinguals, but at similar rates to bilinguals in Vancouver (Byers-Heinlein, 2013) and the U.S. (Place & Hoff, 2016; Potter et al., 2019).

Similar to Study 1, we then examined patterns of language mixing, motivations for mixing and whether they evolve with children’s age, as well as predictors of mixing. Our predictions were unchanged from Study 1.

Lastly, we examined the relationship between parental language mixing and children’s vocabulary size, and approached this question exploratively without strong directional hypotheses.

### 3.2. Methods

This study used archival data collected from children who participated in various experimental studies conducted at the Concordia Infant Research Lab between 2012 and 2019. All studies were approved by the Human Research Ethics Board of Concordia University. While we did not specifically pre-register this study, it was completed after Study 1, and all analyses were conducted in the same manner.

#### Participants

Our sample consisted of 86 heritage-language bilingual families (89 data points as some participants contributed data twice) aged 8–33 months (*M* = 16.68, *SD* = 7.15, 55% female). Participants were recruited using the same procedures as in Study 1, and the inclusion criteria remained unchanged.

Children had at least 10% exposure to their heritage language, and at least 10% exposure to either English or French. We retained participants if they had more than 10% exposure to a third language, if that third language was either English or French (*n* = 22) as frequent exposure to both is common in the Montreal context. On average, participants were exposed to a third language (if any) 6% of the time. Overall, participants received 47% of their language input in their heritage language and 53% in the societal languages (English and French), with 28% exposure to English and 26% to French.^2^ Our sample included participants exposed to 26 different heritage languages. The three most commonly reported were Spanish (35%), Arabic (12%), and Italian (7%), which are the three most common mother tongues in Montreal, besides English and French (Statistics Canada, 2023a). While 46% of parents predominantly spoke the heritage language with their child, 20% predominantly spoke English, 13% predominantly spoke French, and 20% reported speaking an equal amount of their heritage language and English or French (these parents were excluded from analyses involving dominant/non-dominant classifications). Moreover, 45% of parents reported mixing behavior between French and their heritage language and 55% reported mixing behavior between English and their heritage language, based on which societal language they most frequently used alongside the heritage language. On average, parents had a balance score of 28.58 (*SD* = 16.57), computed using the two languages they reported mixing on the LMQ. All participants completed the LEQ, LMQ, and the CDI in either English or French, depending on which language they reported speaking alongside their heritage language. Vocabulary scores for children’s heritage language were not available.

### 3.3. Results

Given that Study 1 already validated the psychometric properties of the Language Mixing Scale in the Montreal context and confirmed its unitary factor structure, we did not repeat the factor analysis for the heritage-language sample. Instead, we proceeded directly to calculate Language Mixing Scale scores as the sum of the five Likert items, consistent with the approach validated in Study 1.

#### 3.3.1. Prevalence of Language Mixing

Parents had a mean mixing score of 14.25 (*SD* = 8.74) out of a possible 30 (see Figure 1B).

Using two-sample, two-tailed *t*-tests, we found that language mixing amongst heritage-language parents in Montreal was not significantly different than Vancouver parents of 18–26-month-old bilingual children (Byers-Heinlein, 2013; *M* = 13.30, *SD* = 7.80; *t*(159) = 0.87, *p* = 0.387, *d* = 0.11); parents of Spanish-English bilingual 30-month-old children in South Florida (Place & Hoff, 2016; *M* = 14.00, *SD* = 7.41; *t*(135) = 0.18, *p* = 0.854, *d* = 0.03); or parents of Spanish-English bilingual 18–30-month-old children in New Jersey (Potter et al., 2019; *M* = 17.00, *SD* = 8.60; *t*(25) = 1.24, *p* = 0.228, *d* = 0.32). However, heritage-language parents in Montreal did mix significantly more than French-English parents in Montreal sampled in Study 1 (*M* = 11.74, *SD* = 7.42; *t*(119) = 2.51, *p* = 0.013, *d* = 0.31; see Figure 2).^3^

These findings indicate that heritage-language bilingual parents in Montreal mix languages significantly more than French-English bilinguals in the same city, whose children were in the same age range. They mixed their languages at similar rates to parents in other heritage-language bilingual communities, although it is possible that differences in the age composition across these samples (i.e., only our heritage language group included younger children of 8–17 months) could influence these results.

#### 3.3.2. Parental Mixing Patterns

We then examined the specific patterns in how parents mixed their languages, focusing on directionality of mixing. Using paired samples *t*-tests, we analyzed whether parents reported different frequencies of mixing depending on the direction of the language switch, both in terms of language dominance and specific languages.

When coded relative to parents’ language dominance, parents did not report switching from their dominant to non-dominant language (*M* = 2.48, *SD* = 2.15) significantly more often than from their non-dominant to dominant language (*M* = 2.41, *SD* = 2.12; *t*(78) = 0.32, *p* = 0.750, *d* = 0.04). Parents also did not report different rates of borrowing words from their dominant language when speaking their non-dominant language (*M* = 2.29, *SD* = 2.15) and borrowing words from their non-dominant language when speaking their dominant language (*M* = 2.61, *SD* = 1.92; *t*(78) = 1.57, *p* = 0.120, *d* = 0.16).

In analyses focused on specific languages rather than dominance, parents again did not report differences in rates of switching to the societal language (i.e., French or English) when speaking their heritage language (*M* = 2.83, *SD* = 2.12) and switching to their heritage language when speaking the societal language (*M* = 2.64, *SD* = 2.17; *t*(101) = 1.02, *p* = 0.312, *d* = 0.09). However, they did report borrowing a societal language word when speaking their heritage language (*M* = 3.08, *SD* = 1.99) significantly more often than borrowing a heritage language word when speaking the societal language (*M* = 2.58, *SD* = 2.10; *t*(101) = 3.65, *p* < 0.001, *d* = 0.24).

Given that Study 1 found parents borrowed English words while speaking French more frequently than the reverse, we conducted further exploratory analyses to compare how often parents borrowed English versus French words. Parents did not report significant differences in borrowing English words (*M* = 3.36, *SD* = 1.89) versus French words (*M* = 2.70, *SD* = 2.09) when speaking their heritage language, *t*(88) = 1.64, *p* = 0.104, *d* = 0.33.

These findings suggest that language dominance does not influence the direction of switches among heritage-language bilinguals in this context. Instead, the societal status of a language appears to shape mixing behavior, with parents more frequently borrowing English or French words when speaking their heritage language than vice versa.

#### 3.3.3. Reasons for Language Mixing and Relations to Child Age

We next examined parents’ motivations for borrowing words from another language. When borrowing from their dominant language while speaking their non-dominant language, the most common reason was uncertainty about the word (47% of parents). When borrowing a word from their non-dominant language while speaking their dominant language, the primary reason was teaching new words (53%).

For language-specific borrowing, 55% of parents reported borrowing a word from their heritage language when speaking the societal language to teach new words, while 52% did so because there was a lack of a suitable translation in French or English. Societal language words were most often borrowed when teaching new words (49%) or due to the uncertainty in translation (45%). See Table 4 for the full proportions of parents reporting each reason for borrowing words.

As in Study 1, the results suggest that parents’ borrowing behavior is shaped by language dominance, with uncertainty about specific words prompting borrowing. Meanwhile, translation gaps and the intention to teach new vocabulary continue to be common motivations for borrowing, regardless of which language the parent is more proficient in.

We then examined if motivations for language mixing might change as children develop by fitting mixed-effects logistic regression models with child age (in years) as a predictor for each potential reason for language mixing. Child age did not predict any of the motivations (all *OR*s < 1.83, *p*s > 0.260). This suggests that heritage-language bilingual parents’ reasons for mixing remain consistent throughout their children’s development, compared to English-French parents who mix more with older children for the purpose of teaching new words.

#### 3.3.4. Predictors of Language Mixing

We next examined the factors that predict the frequency of parental language mixing. Pearson correlations showed that Language Mixing Scale scores were positively correlated with both the parent balance scores (*r* = 0.41, *p* < 0.001) and the number of contexts in which both languages were used (*r* = 0.35, *p* < 0.001). This indicates that higher balance in language use and greater context diversity were associated with more frequent language mixing. No significant correlation was found between language mixing and child age (*r* = 0.10, *p* = 0.331).

To examine the relative contribution of these factors, a multiple linear regression was conducted predicting language mixing from parent balance score, number of contexts, and child age in years (see Table 5). The overall model was significant, *F*(3, 85) = 8.81, *p* < 0.001, with an *R*^2^ of 0.210. Two significant predictors emerged in this multivariate analysis: parents who reported more language mixing were more balanced in their bilingual language output, and spoke their languages in more contexts. However, child age did not significantly predict language mixing when controlling for these other factors.

These findings suggest that the degree to which parents use both languages relatively equally with their child and the number of contexts they use their languages are the strongest predictors of language mixing, similar to Study 1.

#### 3.3.5. Parental Language Mixing’s Relationship to Children’s Vocabulary Size

Finally, we examined the relationship between language mixing and children’s vocabulary development in the societal language. Each child had vocabulary scores reported in either English or French, depending on the language their parents reported mixing with their heritage language on the LMQ. We, thus, ran models on both receptive and productive vocabulary on the subgroup of children learning English, the subgroup of children learning French, and the full sample of children in whichever societal language they were learning. We used a significance threshold of *p* < 0.05 and also applied a Bonferroni correction to account for the multiple comparisons across vocabulary measures.

We first calculated Pearson pairwise correlations between language mixing scores and each vocabulary metric. These analyses revealed no significant association between any of the vocabulary metrics and language mixing, with the exception of English production vocabulary, *r* = 0.31, *p* = 0.032, which showed a weak but statistically significant correlation such that more mixing was associated with a larger vocabulary. However, this correlation would not survive correction for multiple comparisons and does not control for age or language exposure.

Thus, we next conducted linear regression models with each vocabulary size metric as the outcome variable, and predictors including language exposure, language mixing score, age, and their interactions. We initially ran linear mixed-effects models as in Study 1, but encountered convergence issues, likely due to the small number of participants with repeated measures. Therefore, we retained only the most recent data point for participants with multiple entries (*n* = 3) and ran standard linear models instead (See Table S5 in the Supplemental Materials for coefficients of all linear models predicting vocabulary).

For all outcome variables relating to production, there was a significant effect of age such that, controlling for other factors, older children knew more words. Additionally, controlling for other factors, children with greater exposure had larger overall productive vocabularies when English and French scores were combined, though not when English and French scores were analyzed on their own. In contrast, age and language exposure were not significant predictors in any of the comprehension models, which may be due to the smaller subset of participants who provided comprehension scores.

**Figure 4:**
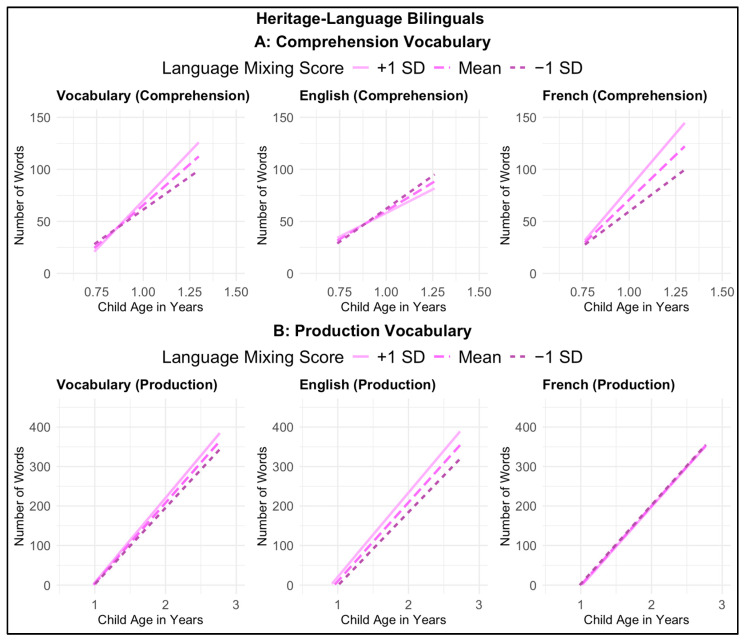
Interaction between child age and language mixing score on vocabulary development among heritage-language bilinguals, for (**A**) comprehension vocabulary measures and (**B**) production vocabulary measures. The x-axes represent child age in years, and the y-axes represent the number of words understood (**A**) or spoken (**B**). Each graph shows predicted vocabulary growth with age for children whose parents’ Language Mixing Scale score was at the sample mean (dashed line), 1 standard deviation above the mean (solid line), and 1 standard deviation below the mean (dotted line).

Most centrally to our research question, no significant effects of or interactions with language mixing were found for any measures of receptive or productive vocabulary (See Figure 4). Our findings suggest that parental language mixing among heritage-language bilinguals is unrelated to children’s vocabulary development, with no consistent positive or negative effects observed across comprehension and production outcomes.

### 3.4. Discussion

Study 2 extended our investigation to heritage-language bilingual families in Montreal to determine whether the patterns observed in French-English bilinguals also apply to other bilingual groups within the same sociolinguistic context. Heritage-language bilinguals mixed languages more frequently than French-English bilinguals, and the directionality of mixing was influenced more by the sociolinguistic status of the language than by language dominance. Additionally, while motivations for mixing were broadly similar across both populations, these motivations did not vary with child age in the heritage-language sample. Mixing was also similarly predicted by greater balance in language use and a wider range of contexts in which both languages were spoken. Finally, similar to the largely null findings in Study 1, we observed no associations between parental language mixing and children’s vocabulary in English or French. Together, these results suggest that parental language mixing is influenced both by the broader sociolinguistic context and by the status of the particular languages involved.

## 4. General Discussion

We investigated the prevalence, predictors, and motivations of parental language mixing among French-English bilinguals (Study 1) and heritage-language bilinguals (Study 2) and its relationship with children’s vocabulary development in a bilingual environment where two languages hold high status. Using parent-reported data from the Language Mixing Questionnaire (Byers-Heinlein, 2013), which we validated in the sample of Montreal French-English bilinguals, we found several key patterns that contribute to our understanding of bilingual language practices.

### 4.1. Language Mixing Rates Vary by Sociolinguistic Context

First, language mixing among French-English bilinguals in Montreal was not statistically different from other contexts with more distinct majority and minority languages, including Vancouver (Byers-Heinlein, 2013) and Florida (Place & Hoff, 2016) after accounting for child age. However, French-English parents did mix less than Spanish-English parents in New Jersey (Potter et al., 2019). We also found that language mixing amongst French-English bilinguals was significantly less frequent than in heritage-language bilinguals in the same city, with both groups of parents reporting on children in the same age range. This notable difference highlights the potential influence of sociolinguistic status on language mixing. Unlike many bilingual settings where a clear societal language dominates, Montreal is a unique community where both French and English hold high status and are frequently encountered in everyday life (Kircher et al., 2022; Leimgruber & Fernández-Mallat, 2021). Research has demonstrated that bilinguals often mix languages to establish community connections and social identity (Botha, 2021; Guerini, 2014; Nilep, 2006; Pérez-Sabater & Moffo, 2019; Phinney et al., 2001). In Montreal’s more balanced bilingual environment, this social-identity function may be less necessary for French-English bilinguals, while remaining important for heritage-language bilinguals, for whom language use can play a central role in framing socio-cultural identity and connecting children to culturally significant knowledge and practices (Curdt-Christiansen, 2009), potentially contributing to the observed difference in mixing rates between French-English and heritage-language bilinguals in the city.

The sociolinguistic history of Quebec may also play a role in shaping language mixing patterns among French-English bilinguals. Historical efforts to preserve French language integrity have promoted attitudes that discourage language mixing (Buoy & Nicoladis, 2018), although research shows that even young adults who express negative views about borrowing English words still engage in the practice (Planchon & Stockemer, 2016, 2020). This complex relationship with language mixing may contribute to the lower rates observed in our French-English sample compared to the heritage-language sample in Montreal.

Moreover, the variable results comparing French-English bilinguals in Montreal to heritage-language bilinguals in other communities suggest that context-specific factors beyond language status may shape parents’ mixing practices. Future studies will be needed to better understand individual and community-level factors that impact parental language mixing.

### 4.2. Directional Patterns Reflect Language Dominance and Status

Our analysis of directional patterns revealed systematic asymmetries in how parents mix their languages. As predicted, French-English parents more frequently borrowed words from their dominant language when speaking their non-dominant language, consistent with cognitive models of bilingual lexical access (Gollan et al., 2014; Liao & Chan, 2016). Interestingly, language-specific analyses showed that parents reported more full-sentence switching from English to French and more word borrowing from English when speaking French. This pattern may reflect the status of certain English loanwords in Montreal French, where terms like “fun,” “coach,” or “condo” have become integrated into everyday usage (Planchon & Stockemer, 2016, 2020). In contrast, the direction of language mixing among heritage-language parents was not associated with language dominance. Instead, parents reported significantly more frequent borrowing of societal language (i.e., French or English) words than heritage language words. Moreover, there were no significant differences in how frequently parents borrowed English compared to French words when speaking their heritage language. This could suggest the influence of societal language prestige and the practical necessity of using terms that are widely recognized and understood within the community. Overall, these findings underscore the importance of considering both language-dominance effects and specific language characteristics when examining bilingual language practices.

### 4.3. Pedagogical Motivations for Language Mixing

When examining parents’ motivations for language mixing, we found that uncertainty about translations was the most common reason cited for borrowing from the dominant language when speaking the non-dominant language across both English-French and heritage-language bilinguals. This aligns with cognitive research showing increased processing demands when operating in a less-dominant language (Liao & Chan, 2016). In Montreal, where both languages are widely used, many French-English parents may maintain relatively balanced proficiency in both languages, potentially reducing the need to mix due to uncertainty, compared to heritage-language bilinguals, where proficiency may be less developed or maintained.

Perhaps most notably, parents frequently reported pedagogical motivations for language mixing. Teaching new vocabulary emerged as a primary reason for borrowing words across languages across both French-English bilingual and heritage bilingual parents, consistent with our prediction and with previous findings (Byers-Heinlein, 2013; Kremin et al., 2022; Nicoladis & Secco, 2000). Additionally, parents of older French-English bilingual children were more likely to report mixing their languages specifically for vocabulary instruction.

Regarding predictors of language mixing, we found that parents with more balanced bilingual output and those who used both languages across diverse contexts reported more frequent mixing in both French-English and heritage-language bilinguals. These relationships align with prior research (Byers-Heinlein, 2013; Verhagen et al., 2022), and reflect natural opportunities for language contact. Child age did not predict language mixing amongst heritage-language parents, suggesting that parents mix consistently throughout their child’s early development. However, in our multivariate analysis, child age emerged as a significant predictor of mixing frequency among French-English parents even after controlling for the balance of bilingual output and context diversity. This finding corroborates our observation that parents of older French-English bilingual children more frequently use language mixing as a teaching strategy, also seen in Kremin et al. (2022). This age-related pattern suggests that an important portion of parental language mixing may represent a deliberate strategy that differs according to children’s developmental stage rather than merely reflecting parents’ linguistic limitations or social habits relative to other bilingual adults. Parents appear to modify their language practices based on their child’s age, potentially to optimize input quality for different developmental stages (Bergelson, 2020; M. L. Rowe & Snow, 2019). However, given that experimental and book-reading studies have found mixed results on the effectiveness of using language mixing to teach new words (Brouillard et al., 2022; Byers-Heinlein et al., 2017, 2022; Kaushanskaya et al., 2022; Libersky et al., 2025; Read et al., 2021), future research should explore the specific conditions under which language mixing serves as a beneficial pedagogical tool versus when it may introduce processing costs.

### 4.4. Limited Associations with Vocabulary Outcomes

We found little evidence for an association between parental language mixing and vocabulary development among French-English and heritage-language bilingual children. Amongst French-English bilinguals, language mixing was associated with greater English production vocabulary, but smaller French production vocabulary, although these effects were modest, and no significant effects were found for the additional 10 models we ran. Among heritage-language bilinguals, we found no significant effects or interactions in any model. Overall, the preponderance of null results and the mixed directionality of the two significant effects we found suggest that parental language mixing is unlikely to be associated with vocabulary development. Traditional predictors such as age and language exposure remained more robust overall.

Our findings have important implications for parents and practitioners concerned about potential negative effects of language mixing on children’s language development. Our results indicate that parental mixing does not appear to cause confusion or impede vocabulary acquisition in this bilingual environment, challenging common misconceptions that have been documented in some communities (Ballinger et al., 2020; Mata, 2023). Ultimately, our results replicate several other studies that have reported null relationships between mixing and vocabulary outcomes (Place & Hoff, 2016; Verhagen et al., 2022; Verhoeven et al., 2025), but are inconsistent with those that have shown positive (Bail et al., 2015), and negative relationships (Byers-Heinlein, 2013; Place & Hoff, 2016; Verhoeven et al., 2025). More research is necessary to understand whether these divergent findings reflect real differences across the populations studied, or are primarily due to methodological factors such as sampling error, measurement differences, or study design variations.

### 4.5. Limitations and Future Directions

While this study provides valuable insights into parental language mixing in a bilingual context with two societal languages, there are several limitations that should be addressed in future research. One limitation is our use of parent-report data to assess language mixing, rather than direct observational measures. While this allowed us to have a larger sample, parent estimates may not always reflect actual input (Marchman et al., 2017) and questionnaires can limit the nuance of data collected. However, our findings regarding the rates and motivations of parental language mixing among French-English bilinguals are consistent with past work in Montreal using home recordings (Kremin et al., 2022), suggesting that parent report measures can still provide meaningful insights.

Next, our operationalization of parents’ language dominance was based on parents’ reported language use with their child rather than direct measures of proficiency. Although the language parents speak most frequently with their children is likely to be their most proficient language, this may not always be the case, and more comprehensive assessments of language proficiency would strengthen future analyses of directional mixing patterns.

An important question for future research is whether the reasons or motivations behind language mixing affect its usefulness for children’s language development. Our findings showed that parents mix languages for diverse reasons, including uncertainty about translations, lexical gaps, and intentional vocabulary teaching. These different types of mixing may have distinct impacts on children’s language learning. For instance, when a parent mixes languages to deliberately teach a new word, they may provide additional scaffolding, emphasis, or contextual support compared to when they mix due to momentary word-finding difficulties. Children might also be more attentive to pedagogically motivated mixing or better able to extract the intended learning target. Understanding these nuanced differences could help explain the mixed findings in the literature regarding language mixing and vocabulary development. Experimental studies that systematically manipulate both the form of language mixing (e.g., borrowing versus sentence-level switching) and its function (e.g., pedagogical versus necessity-driven) would help clarify when and how mixed language input facilitates or challenges children’s language acquisition.

This study also did not directly assess parents’ attitudes towards language mixing, which could influence both the frequency of mixing and its impact on children’s language development. Parents’ beliefs about the benefits or drawbacks of mixing languages—whether they see it as a positive strategy for teaching their child or as a potential source of confusion—may shape how and when they mix languages. Attitudes towards language mixing may differ across bilingual groups—for example, French-English households and heritage-language households in Quebec hold different attitudes towards childhood multilingualism (Kircher et al., 2022). Future research could explore the role of parents’ attitudes to better understand how these beliefs align with their language practices and how they affect children’s vocabulary outcomes, especially across different types of bilingual families.

Finally, our studies only examined French and English vocabulary development, as we did not have data on heritage language vocabulary size. Future work should examine vocabulary development in the heritage language, rather than focusing solely on societal languages. Some prior work has found negative relationships between parental language mixing and children’s heritage language outcomes (Place & Hoff, 2016; Verhoeven et al., 2025). Thus, it is possible that parental language mixing influences heritage-language development differently than societal-language development. Understanding whether and how mixing supports or hinders vocabulary growth in the heritage language is essential, particularly for efforts to support heritage language maintenance in multilingual families.

## 5. Conclusions

Overall, the findings from these studies suggest that parental language mixing reflects adaptive communication strategies rather than detrimental practices. The sociolinguistic context of Montreal appears to influence both the frequency and functions of language mixing in parent–child interactions. Our results support the view that bilingual children can successfully navigate mixed language input, and that parents adjust their mixing patterns according to children’s developmental stage. This research advances our understanding of bilingual language development in diverse contexts and provides evidence-based insights for bilingual families navigating multiple languages during early childhood.

## Figures and Tables

**Table 1:** Descriptive statistics, factor loadings, and inter-item Spearman’s rank correlations for dominant vs. non-dominant coding.

Variable	M	SD	Factor Loading	Inter-Item Correlations				
				Switch Dom → NonDom	Switch NonDom → Dom	Borrow Dom Word	Borrow NonDom Word	Mix Both Languages
Switch Dom → NonDom	1.93	1.81	0.77	–				
Switch NonDom → Dom	2.10	1.83	0.65	0.59 ***	–			
Borrow Dom Word	2.64	1.98	0.66	0.44 ***	0.45 ***	–		
Borrow NonDom Word	2.32	1.84	0.74	0.62 ***	0.39 ***	0.54 ***	–	
Mix Both Languages	2.60	1.93	0.75	0.61 ***	0.52 ***	0.55 ***	0.53 ***	–

Note. Switch Dom → NonDom = “I often start a sentence in Dominant language and then switch to speaking Non-dominant language”; Switch NonDom → Dom = “I often start a sentence in Non-dominant language and then switch to Dominant language”; Borrow Dom Word = “I often borrow a Dominant language word when speaking Non-dominant language”; Borrow NonDom Word = “I often borrow a Non-dominant language word when speaking Dominant language”; Mix Both Languages = “In general, I often mix English and French”; *** *p* < 0.001.

**Table 2:** Reasons French-English bilingual parents reported for borrowing words from another language (reported as proportion of parents who provided a response).

	Borrowing Dominant Language Word When Speaking Non-Dominant Language	Borrowing Non-Dominant Language Word When Speaking Dominant Language	Borrowing English Word When Speaking French	Borrowing French Word When Speaking English
I’m not sure of the word	0.63	0.28	0.49	0.39
No translation or only a poor translation exists for the word	0.47	0.53	0.51	0.48
The word is hard to pronounce	0.27	0.13	0.22	0.16
When I’m teaching new words	0.42	0.53	0.44	0.50
Other times/not sure	0.20	0.32	0.27	0.28

Note. Parents could select multiple reasons for borrowing words, so values within each column do not sum to 1.00. Reasons were coded in two ways: for English vs. French words and for dominant vs. non-dominant language words.

**Table 3:** Summary of linear regression model predicting language mixing score among French-English bilingual parents.

Parameter	Unstandardized Estimate (*B*)	*SE*	Standardized Estimate (β)	*t*(385)	*p*
Intercept	3.58	1.69	0.66	2.12	0.035
Parent balance score	0.23	0.03	0.04	8.95	<0.001
Number of contexts	0.17	0.17	0.03	1	0.319
Child age in years	1.46	0.56	0.27	2.62	0.009

**Table 4:** Reasons heritage-language bilingual parents reported for borrowing words from another language (reported as proportion of parents who provided a response).

	Borrowing Dominant Language Word When Speaking Non-Dominant Language	Borrowing Non-Dominant Language Word When Speaking Dominant Language	Borrowing Heritage Language Word When Speaking Societal Language	Borrowing Societal Language Word When Speaking Heritage Language
I’m not sure of the word	0.47	0.25	0.22	0.45
No translation or only a poor translation exists for the word	0.41	0.37	0.52	0.39
The word is hard to pronounce	0.31	0.25	0.23	0.34
When I’m teaching new words	0.45	0.53	0.55	0.49
Other times/not sure	0.31	0.28	0.29	0.24

Note. Parents could select multiple reasons for borrowing words, so values within each column do not sum to 1.00. Reasons were coded in two ways: for societal language vs. heritage language words and for dominant vs. non-dominant language words.

**Table 5:** Summary of linear regression model predicting language mixing score among heritage-language bilingual parents.

	Unstandardized Estimate (*B*)	*SE*	Standardized Estimate (β)	*t*(85)	*p*
Intercept	0.30	3.36	0.05	0.09	0.929
Parent balance score	0.18	0.05	0.03	3.50	0.001
Number of contexts	0.86	0.36	0.14	2.37	0.020
Child age in years	1.60	1.40	0.26	1.15	0.253

## Data Availability

All data and code is publicly available through an OSF repository: https://osf.io/3g6ze/ (accessed on 15 September 2025).

## Notes

1 Percentages do not add up to 100 due to rounding.

2 Percentages do not add up to 53 due to rounding.

3 While we restricted the sample in Study 1 to parents of 18–30-month-olds to account for potential age differences with comparison groups, we were unable to do so in Study 2 due to the smaller sample size.

## Supplementary Materials

The following supporting information can be downloaded at: https://www.mdpi.com/article/10.3390/bs15101371/s1, Table S1: Descriptive statistics, factor loadings, and inter-item Pearson’s correlations for dominant vs. non-dominant coding; Table S2: Descriptive statistics, factor loadings, and inter-item Spearman’s rank correlations for French vs. English coding; Table S3: Descriptive statistics, factor loadings, and inter-item Pearson’s correlations for French vs. English coding; Table S4: Coefficient estimates from linear mixed-effects models predicting vocabulary size among French-English bilinguals; Table S5: Coefficient estimates from linear models predicting vocabulary size among heritage-language bilinguals.
