# Supplementary material for: Parental Language Mixing in Montreal: Rates, Predictors, and Relation to Infants’ Vocabulary Size
Source: Behav Sci (Basel). 2025 Oct 8;15(10):1371. doi: 10.3390/bs15101371 (PMC12561030; doi:10.3390/bs15101371)
Supplement: Supplementary file 1 [file behavsci-15-01371-s001.zip › behavsci-3778685-supplementary.pdf]

Supplemental Materials

Deviations from Pre-Registration of Study 1

We deviated from our pre-registered plan in the following:

First, our final sample size was reduced by one participant after we identified that a parent reported mixing between languages other than French and English.

Second, as pre-registered, we examined whether the five Likert-scale items on the Language Mixing Questionnaire (LMQ) reflected a unitary construct by performing correlations and conducting an exploratory factor analysis. Based on a reviewer's suggestion, we also reported Spearman's rank correlations for the Likert-scale responses in the main manuscript, rather than the pre-registered Pearson's correlations which we now report here (Tables S2 and S3), although we note that the values were extremely similar.

Third, rather than performing the pre-registered chi-square goodness-of-fit test reported in Byers-Heinlein (2013), we conducted a parallel analysis using R for the dominant/non-dominant coding due to software constraints. The results of the exploratory factor analysis (EFA) clearly indicated a one-factor solution for both dominant/non-dominant and French/English coding, making further analyses in this direction unnecessary.

Fourth, we modified our comparative analysis with the sample from Place and Hoff (2016). We initially pre-registered a comparison with 38 mothers of 30-month-old Spanish-English bilinguals in the U.S. (M = 15.26, SD = 7.00). However, we subsequently determined that this was only a subset of the study participants. Our analyses instead compared our results to the full sample of 58 mothers who completed the Language Mixing Questionnaire (LMQ) in that study, which reported a mean language mixing score of 14.00 (SD = 7.41).

Finally, we expanded our analytical approach beyond the pre-registered analyses of mixing patterns between the dominant and non-dominant languages. We added analyses examining French/English mixing to fully explore whether characteristics inherent to these particular languages influenced the direction and patterns of language mixing.

Psychometric Properties of the Language Mixing Scale

Dominant vs. non-dominant language coding

To remain consistent with our pre-registration, we also report Pearson's correlations among the five Language Mixing Questionnaire (LMQ) items in addition to the Spearman's rank correlations reported in the main manuscript for the dominant vs. non-dominant coding. All five items were significantly intercorrelated (rs = 0.40–0.61, all ps < .001). An exploratory factor analysis examining the scale's underlying factor structure revealed a primary component with an eigenvalue of 3.05, accounting for 51% of the variance.

Supplemental Table S1. Descriptive statistics, factor loadings, and inter-item Pearson's correlations for dominant vs. non-dominant coding.

Variable	M	SD	Factor Loading	Inter-Item Correlations				
				Switch Dom → NonDom	Switch NonDom → Dom	Borrow Dom Word	Borrow NonDom Word	Mix Both Languages
Switch Dom → NonDom	1.93	1.81	.77	–				
Switch NonDom → Dom	2.10	1.83	.65	.54***	–			
Borrow Dom Word	2.64	1.98	.66	.42***	.47***	–		
Borrow NonDom Word	2.32	1.84	.74	.61***	.40***	.54***	–	
Mix Both Languages	2.60	1.93	.75	.58***	.51***	.53***	.53***	–

Note. Switch Dom → NonDom = "I often start a sentence in Dominant language and then switch to speaking Non-dominant language"; Switch NonDom → Dom = "I often start a sentence in Non-dominant language and then switch to Dominant language"; Borrow Dom Word = "I often borrow a Dominant language word when speaking Non-dominant language"; Borrow NonDom Word = "I often borrow a Non-dominant language word when speaking Dominant language"; Mix Both Languages = "In general, I often mix English and French"; ***p < .001

English vs. French coding

Spearman's rank correlations between all items were significant (rs = 0.36–0.61, all ps < .001). An exploratory factor analysis was done to examine the underlying factor structure of the scale. The first

component extracted had an eigenvalue of 3.11 which accounted for 52% of the variance. All other eigenvalues were under 1, suggesting a one-factor solution. Inter-item Spearman's rank correlations and extracted loadings of all the items on the factor are reported in Supplemental Table S2. The scale demonstrated good internal consistency, with a Cronbach's alpha of α = 0.84.

Supplemental Table S2. Descriptive statistics, factor loadings, and inter-item Spearman's rank correlations for French vs. English coding.

**Variable**	***M***	***SD***	**Factor Loading**	**Inter-Item Correlations**				
				**Switch En → Fr**	**Switch Fr → En**	**Borrow En Word**	**Borrow Fr Word**	**Mix Both Languages**
**Switch En → Fr**	2.23	1.89	.73	–				
**Switch Fr → En**	1.89	1.76	.72	.61***	–			
**Borrow En Word**	2.59	1.94	.67	.36***	.50***	–		
**Borrow Fr Word**	2.38	1.90	.73	.57***	.46***	.55***	–	
**Mix Both Languages**	2.64	1.96	.76	.59***	.55***	.54***	.54***	–

Note. Switch En → Fr = "I often start a sentence in English language and then switch to speaking French"; Switch Fr → En = "I often start a sentence in French language and then switch to English"; Borrow En Word = "I often borrow an English word when speaking French"; Borrow Fr Word = "I often borrow a French word when speaking English"; Mix Both Languages = "In general, I often mix English and French"; ***p<.001

Again, to remain consistent with our pre-registration, we also report Pearson's correlations among the five LMQ items. Pearson's correlations between all items were significant (rs = 0.36–0.58, all ps < .001). An exploratory factor analysis was done to examine the underlying factor structure of the scale. The first component extracted had an eigenvalue of 3.08 which accounted for 52% of the variance. All other eigenvalues were under 1, suggesting a one-factor solution. Inter-item Pearson's correlations and extracted loadings of all the items on the factor are reported in Supplemental Table S3.

Supplemental Table S3. Descriptive statistics, factor loadings, and inter-item Pearson's correlations for French vs. English coding.

**Variable**	***M***	***SD***	**Factor Loading**	**Inter-Item Correlations**				
				**Switch En → Fr**	**Switch Fr → En**	**Borrow En Word**	**Borrow Fr Word**	**Mix Both Language s**
**Switch En → Fr**	2.23	1.89	.73	–				
**Switch Fr → En**	1.89	1.76	.72	.58***	–			
**Borrow En Word**	2.59	1.94	.67	.36***	.52***	–		
**Borrow Fr Word**	2.38	1.90	.73	.57***	.46***	.55***	–	
**Mix Both Languages**	2.64	1.96	.76	.57***	.53***	.53***	.53***	**–**

Note. Switch En → Fr = "I often start a sentence in English language and then switch to speaking French"; Switch Fr → En = "I often start a sentence in French language and then switch to English"; Borrow En Word = "I often borrow an English word when speaking French"; Borrow Fr Word = "I often borrow a French word when speaking English"; Mix Both Languages = "In general, I often mix English and French"; ****p*<.01

Linear Mixed Effects Models Predicting Vocabulary in French-English Bilinguals

Supplemental Table S4. Coefficient estimates from linear mixed-effects models predicting vocabulary size among French-English bilinguals.

Model	Parameter	Unstandardized Estimate (*B*)	*SE*	Standardized Estimate (β)	*t*	*df*	*p*
Word Production	(Intercept)	-339.95	42.65	-1.71	-7.97	383	<.001
	lang_mixing_score	1.15	3.12	0.01	0.37	383	0.713
	child_age_in_years	319.96	24.21	1.61	13.22	383	<.001
	lang_mixing_score: child_age_in_years	-0.65	1.74	0	-0.38	383	0.707
Word Comprehension	(Intercept)	-55.34	82.18	-1.42	-0.67	149	0.502
	lang_mixing_score	4.8	5.97	0.12	0.8	149	0.423
	child_age_in_years	192.31	74.23	4.93	2.59	149	0.011
	lang_mixing_score: child_age_in_years	-4.48	5.31	-0.11	-0.84	149	0.4
Concept Production	(Intercept)	-260.3	29.97	-1.64	-8.68	383	<.001
	lang_mixing_score	0.21	2.19	0	0.09	383	0.924
	child_age_in_years	248.62	16.97	1.56	14.65	383	<.001
	lang_mixing_score: child_age_in_years	-0.12	1.21	0	-0.1	383	0.919
Concept Comprehension	(Intercept)	-54.09	56.02	-1.57	-0.97	149	0.336
	lang_mixing_score	4.21	4.08	0.12	1.03	149	0.304
	child_age_in_years	160.81	50.53	4.66	3.18	149	0.002
	lang_mixing_score: child_age_in_years	-4.05	3.62	-0.12	-1.12	149	0.265
Dominant Production	(Intercept)	-326.52	49.86	-2.25	-6.55	378	<.001
	lang_mixing_score	1.91	2.24	0.01	0.85	378	0.394
	child_age_in_years	237.95	17.37	1.64	13.7	378	<.001
	exposure_to_dominant_lang	101.49	61.12	0.7	1.66	378	0.098

	lang_mixing_score: child_age_in_years	-1.15	1.25	-0.01	-0.92	378	0.356
Dominant Comprehension	(Intercept)	-87.82	59.59	-2.93	-1.47	145	0.143
	lang_mixing_score	2.45	3.72	0.08	0.66	145	0.512
	child_age_in_years	131.43	46.85	4.38	2.81	145	0.006
	exposure_to_dominant_lang	69.76	60.83	2.33	1.15	145	0.253
	lang_mixing_score: child_age_in_years	-2.74	3.31	-0.09	-0.83	145	0.409
Non-Dominant Production	(Intercept)	-157.08	25.12	-2.69	-6.25	378	<.001
	lang_mixing_score	0.21	1.53	0	0.14	378	0.889
	child_age_in_years	86.2	11.87	1.48	7.26	378	<.001
	exposure_to_nondominant_lang	209.93	39.83	3.6	5.27	378	<.001
	lang_mixing_score: child_age_in_years	-0.07	0.86	0	-0.08	378	0.936
Non-Dominant Comprehension	(Intercept)	-85.39	45.88	-3.91	-1.86	145	0.065
	lang_mixing_score	3.24	2.9	0.15	1.12	145	0.265
	child_age_in_years	73.74	36.42	3.38	2.02	145	0.045
	exposure_to_nondominant_lang	172.8	48.04	7.91	3.6	145	<.001
	lang_mixing_score: child_age_in_years	-2.64	2.58	-0.12	-1.02	145	0.308
English Production	(Intercept)	-264.39	34.31	-2.05	-7.71	382	<.001
	lang_mixing_score	-3.41	2.23	-0.03	-1.53	382	0.128
	child_age_in_years	152.21	17.32	1.18	8.79	382	<.001
	exposure_to_english	201.28	29.7	1.56	6.78	382	<.001
	lang_mixing_score: child_age_in_years	2.55	1.24	0.02	2.05	382	0.041
English Comprehension	(Intercept)	-60.18	49.37	-1.89	-1.22	148	0.225
	lang_mixing_score	0.03	3.47	0	0.01	148	0.993

	child_age_in_years	58.13	43.17	1.82	1.35	148	0.18
	exposure_to_english	128.8	27.52	4.04	4.68	148	<.001
	lang_mixing_score: child_age_in_years	0.45	3.09	0.01	0.15	148	0.883
French Production	(Intercept)	-272.41	26.75	-2.9	-10.18	382	<.001
	lang_mixing_score	4.3	1.8	0.05	2.39	382	0.017
	child_age_in_years	165.5	13.91	1.76	11.9	382	<.001
	exposure_to_french	204.17	23.23	2.17	8.79	382	<.001
	lang_mixing_score: child_age_in_years	-3.1	1	-0.03	-3.09	382	0.002
French Comprehension	(Intercept)	-116.66	47.15	-3.38	-2.47	148	0.014
	lang_mixing_score	4.86	3.28	0.14	1.48	148	0.141
	child_age_in_years	136.11	40.61	3.94	3.35	148	0.001
	exposure_to_french	108.68	26.23	3.15	4.14	148	<.001
	lang_mixing_score: child_age_in_years	-5	2.91	-0.14	-1.72	148	0.088

Linear Models Predicting Vocabulary in Heritage-Language Bilinguals

Supplemental Table S5. Coefficient estimates from linear models predicting vocabulary size among heritage-language bilinguals.

Model	Parameter	Unstandardized Estimate (*B*)	*SE*	Standardized Estimate (β)	*t*	*df*	*p*
Production	(Intercept)	-232.02	50.18	-1.79	-4.62	81	<.001
	lang_mixing_score	-0.98	2.91	-0.01	-0.34	81	0.738
	child_age_in_years	186.23	32.91	1.44	5.66	81	<.001
	exposure_to_form_ language	98.5	46.2	0.76	2.13	81	0.036
	lang_mixing_score: child_age_in_years	1.22	1.91	0.01	0.64	81	0.524
Comprehension	(Intercept)	-80.5	86.88	-2.38	-0.93	53	0.358
	lang_mixing_score	-3.07	5.46	-0.09	-0.56	53	0.577
	child_age_in_years	108.84	79.22	3.22	1.37	53	0.175
	exposure_to_form_ language	66.55	39.42	1.97	1.69	53	0.097
	lang_mixing_score: child_age_in_years	3.58	5.03	0.11	0.71	53	0.48
English Production	(Intercept)	-218.71	81.78	-1.71	-2.67	42	0.011
	lang_mixing_score	-0.33	5.04	0	-0.07	42	0.948
	child_age_in_years	174.78	59.13	1.37	2.96	42	0.005
	exposure_to_form_ language	71.52	76.64	0.56	0.93	42	0.356
	lang_mixing_score: child_age_in_years	1.67	3.24	0.01	0.52	42	0.609
English Comprehension	(Intercept)	-88.97	112.9	-3.96	-0.79	23	0.439
	lang_mixing_score	2	8.32	0.09	0.24	23	0.812
	child_age_in_years	136.36	106.33	6.08	1.28	23	0.212
	exposure_to_form_ language	34.94	62.64	1.56	0.56	23	0.582

	lang_mixing_score: child_age_in_years	-2.26	7.87	-0.1	-0.29	23	0.777
French Production	(Intercept)	-246.2	63.37	-1.85	-3.88	34	<.001
	lang_mixing_score	-0.45	3.53	0	-0.13	34	0.9
	child_age_in_years	198.76	37.33	1.49	5.32	34	<.001
	exposure_to_form_ language	112.09	57.87	0.84	1.94	34	0.061
	lang_mixing_score: child_age_in_years	0.09	2.38	0	0.04	34	0.968
French Comprehension	(Intercept)	-107.15	149.65	-2.42	-0.72	25	0.481
	lang_mixing_score	-2.98	8.57	-0.07	-0.35	25	0.731
	child_age_in_years	111.06	130.73	2.51	0.85	25	0.404
	exposure_to_form_ language	105.78	58.92	2.39	1.8	25	0.085
	lang_mixing_score: child_age_in_years	4.23	7.67	0.1	0.55	25	0.586
